# Differential in vitro inhibitory activity against HIV-1 of alpha-(1-3)- and alpha-(1-6)-D-mannose specific plant lectins : Implication for microbicide development

**DOI:** 10.1186/1479-5876-5-28

**Published:** 2007-06-12

**Authors:** Hela Saïdi, Nadine Nasreddine, Mohammad-Ali Jenabian, Maxime Lecerf, Dominique Schols, Corinne Krief, Jan Balzarini, Laurent Bélec

**Affiliations:** 1 Unité INSERM U743, Equipe « Immunité et Biothérapie Muqueuse », Centre de Recherches Biomédicales des Cordeliers, Paris, France; 2 Rega Institute for Medical Research, Leuven, Belgium

## Abstract

**Background:**

Plant lectins such as *Galanthus nivalis *agglutinin (GNA) and *Hippeastrum hybrid *agglutinin (HHA) are natural proteins able to link mannose residues, and therefore inhibit HIV-target cell interactions. Plant lectins are candidate for microbicide development.

**Objective:**

To evaluate the activity against HIV of the mannose-specific plant lectins HHA and GNA at the cellular membrane level of epithelial cells and monocyte-derived dendritic cells (MDDC), two potential target cells of HIV at the genital mucosal level.

**Methods:**

The inhibitory effects of HHA and GNA were evaluated on HIV adsorption to genital epithelial HEC-1A cell line, on HIV transcytosis throughout a monolayer of polarized epithelial HEC-1A cells, on HIV adsorption to MDDC and on transfer of HIV from MDDC to autologous T lymphocytes.

**Results:**

HHA faintly inhibited attachment to HEC-1A cells of the R5-tropic HIV-1_Ba-L _strain, in a dose-dependent manner, whereas GNA moderately inhibited HIV adsorption in the same context, but only at high drug doses. Only HHA, but not GNA, inhibited HIV-1_JR-CSF _transcytosis in a dose-dependent manner. By confocal microscopy, HHA, but not GNA, was adsorbed at the epithelial cell surface, suggesting that HHA interacts specifically with receptors mediating HIV-1 transcytosis. Both plant lectins partially inhibited HIV attachment to MDDC. HHA inhibited more efficiently the transfer of HIV from MDDC to T cell, than GNA. Both HHA and GNA lacked toxicity below 200 μg/ml irrespective the cellular system used and do not disturb the monolayer integrity of epithelial cells.

**Conclusion:**

These observations demonstrate higher inhibitory activities of the lectin plant HHA by comparison to GNA, on HIV adsorption to HEC-1A cell line, HIV transcytosis through HEC-1A cell line monolayer, HIV adsorption to MDDC and HIV transfer from MDDC to T cells, highlighting the potential interest of HHA as effective microbicide against HIV.

## Background

Heterosexual contact is the primary mode of human immunodeficiency virus type 1 (HIV-1) transmission worldwide. The majority of new HIV-1-infected people are women. Effective topical microbicides, as molecules with potent activity against HIV-1, inserted into the vagina prior to sexual intercourse, would offer an alternative to condom use that would be controlled by the receptive partner [1].

Plant lectins represent a well-defined class of antiretroviral compounds that are most advantageous than other antiviral drugs: lectins are natural and not synthetic products (proteins) and target the sugar moieties of a wide variety of glycoproteins [2,3]. *Galanthus nivalis *agglutinin (Snowdrop) (GNA) has specificity for terminal α(1–3)-linked mannose residues, whereas *Hippeastrum hybrid *agglutinin (Amaryllis) (HHA) recognizes both terminal and internal α(1–3)- and α(1–6)-linked mannose residues [4]. These lectins occur as tetramers exhibiting a molecular mass of 50 kDa. They are potent and highly selective inhibitors of the spread of HIV by virus- to- lymphocytes, as well as infected lymphocytes- to- uninfected lymphocytes. HHA and GNA selectively inhibited a wide variety of HIV-1 and HIV-2 strains and clinical (CXCR4- and CCR5-using) isolates in different cell types [4]. It was also shown that these lectins inhibited infection of T lymphocytes by a variety of mutant virus strains, but also prevented syncytium formation between persistently HIV-infected cells and uninfected T lymphocytes [4]. There is strong evidence that plant lectins with anti-HIV activity predominantly target the heavily glycosylated gp120 envelope glycoprotein, as assessed by HIV-lectins pre-incubation assays [4]. In contrast, no marked lectin-lymphocyte interactions were observed [4]. In addition, HHA and GNA efficiently abrogated the DC-SIGN-directed HIV-1 capture in DC-SIGN-expressing B-lymphoblast Raji cells (Raji/DC-SIGN) and its subsequent transfer to CD4^+ ^T lymphocytes [5]. HHA and GNA are also perfectly fitted to microbicide guidelines in the way they are odorless, tasteless, colorless, not mitogenic and not anti-metabolically active at anti-viral concentrations [4].

Since early microbicide trials raised concerns about testing incompletely characterized compounds in humans [6], we propose to complete the *in vitro *pre-clinical evaluation of microbicide molecule candidates against HIV-1 adsorption on epithelial cells, and against HIV-1 transcytosis through a tight monolayer of epithelial cells used as an *in vitro *model mimicking the penetration of HIV-1 through unstratified epithelia [7]. Indeed, epithelial cells represent the most important interface between host and environment. Hocini *et al*. have previously hypothesized that simple epithelial monolayers, such as the endocervix and rectal mucosae, may support rapid transcellular transport (or " transcytosis ") from the apical to basolateral side of the cell, and thus provide viral access to the target cells of the submucosa, such as dendritic cells, CD4^+ ^T cells and macrophages [8].

In the present study, we thus aimed at characterizing whether interactions exist between mannose-specific lectins and mannosylated HIV-interacting molecules. Indeed, HIV was described to interact with a large number of molecules onto cell surface during the adsorption phase. If some of these receptors are mannosylated close to the HIV-binding sites, the plant lectins may recognise such mannosylated residues, and may mask the HIV binding site by steric hindrance, thus limiting HIV attachment onto cells. The effects of carbohydrate-binding plant lectins on T lymphocytes [4] and on DC-SIGN^+ ^B-lymphoblast Raji cells [5] have been previously investigated. To complete these latter studies, we herein evaluated the inhibitory effect of HHA and GNA on the attachment of HIV on other possible mucosal target cells, including epithelial and dendritic cells.

## Methods

### Cells lines, virus strains and plant lectins

the epithelial cell line HEC-1A was maintained in RPMI 1640 containing 10% FCS and antibiotics (100 μg of streptomycin per ml, 100 IU of penicillin per ml). Primary HIV-1_NDK _X4-tropic viruses (gift from Prof. F. Barré-Sinoussi, Institut Pasteur, Paris, France) were grown in peripheral blood lymphocytes (PBL) of healthy donors stimulated with phytohemagglutinin-P (PHA; Sigma-Aldrich, Saint Louis, MO) and interleukin-2 (IL-2; Peprotech Inc, Rocky Hill, NJ). R5-tropic HIV-1_J-RCSF _and HIV-1_Ba-L _were amplified in monocyte-derived macrophages (MDM) of healthy donors. Tropism of viruses was determined using U87 cells (provided by Dr. E. Menue, Institut Pasteur) transfected with DNA encoding for human CD4, CCR5, or CXCR4. HIV was quantified in cell culture supernatants by means of the DuPont HIV-p24 antigen ELISA (HIV-1 core profile ELISA; DuPont de Nemours, Les Ulis, France). The mannose-specific lectins HHA and GNA were derived and purified from the bulbs of these plants, as previously described [9]. Mannan was provided by Sigma.

### *In vitro *differentiation of monocyte-derived dendritic cells (MDDC)

MDDC were generated from monocytes as previously described. Briefly, peripheral blood mononuclear cells (PBMC) were isolated from buffy coats of healthy adult donors by Ficoll density gradient centrifugation on MSL (Medium for Separate of Lymphocytes, Eurobio, Les Ulis, France). The percentage of monocytes was determined by flow cytometry on a FACScalibur (Becton Dickinson, Montain View, CA) using forward scatter and side scatter properties (FSC/SSC). PBMC were re-suspended in RPMI 1640medium supplemented with L-glutamine (BioWhittaker Europe, Verviers, Belgium), penicillin (100 IU/ml) and streptomycin (100 μg/ml) (Gibco BRL, Paisley, Scotland). Cells were seeded into 24 well-plates (Costar, Cambridge, MA) at the concentration 1 × 10^6 ^adherent cells/ml. Cells were incubated at 37°C for 45 min. Non-adherent cells were removed by 4 gently washes. Adherent monocytes were further incubated in RPMI medium supplemented with 10% fetal calf serum (FCS), L-glutamine, and antibiotics in the presence of 10 ng/ml rhIL-4 and 10 ng/ml rhGM-CSF (both from R&D Systems, Oxon, UK), in order to differentiate MDDC. Half of the medium, including all supplements, was replaced every 2 days. After 6 days of culture, non-adherent cells corresponding to the DC-enriched fraction were harvested, washed, and used for subsequent experiments. Flow cytometry analysis demonstrated that the DC were 90% or more pure DC at an immature stage (CD14^-^, CD16^-^, CD1a^+^, CD83^-^, DC-SIGN^+^).

### Isolation of autologous T lymphocytes

PBL were subsequently prepared from the monocyte-depleted cell fraction. PBL were cultured for 48 h in fresh medium supplemented with PHA (2.5 μg/ml) and IL-2 (1 μg/ml). PBL were then washed and further cultured in growth medium containing IL-2 (1 μg/ml) for 24 h.

### Cytotoxicity assay

the cytotoxicity of the plant lectins against epithelial cells and MDDC was analysed using the MTT (3- [4,5-dimethylthiazol-2-yl]-2,5-diphenyl tetrazolium bromide) assay (Sigma). Briefly, cells were seeded onto 96-well plates at a density of 2 × 10^5 ^cells/well and incubated for 24 h at 37°C prior to drug exposure. On the day of treatment, culture medium was carefully aspirated from the wells and replaced with fresh medium containing the plant lectins at concentrations ranging from 1 to 500 μg/ml. Triplicate wells were used for each treatment. The cells were incubated with the various compounds for 24 h at 37°C in a humidified 5% CO2 atmosphere. To each well, 20 μl of MTT (0.5 mg/ml final concentration) was added and the plates were incubated at 37°C for 4 h to allow MTT to form formazan crystals by reacting with metabolically active cells. The formazan crystals were solubilized 30 min at 37°C in a solution containing 10% sodium dodecyl sulphate in 0.01 M HCl. The absorbance of each well was measured in a microtitre reader at 490 nm. To translate the OD_490 _values into the number of live cells in each well, the OD_490 _values were compared with those of standard OD_490 _*versus *cell number curves generated for each cell type. The percentage survival was calculated using the formula :

% survival = live cell number (test)/live cell number (control) × 100

### Epithelial monolayer integrity

the effect of the plant lectins on their ability to maintain an intact epithelium was determined by measuring transepithelial resistance (TER). HEC-1A cells were grown as a tight polarized monolayer on a permeable support of 0.4 μm-pore-diameter polycarbonate (Transwell, Costar, Cambridge, Mass.). Apical and basolateral media were replaced, and TER was measured daily with a Millicell-ERS (electrical resistance system) instrument (Millipore Corporation, Bedford, Mass.). When plateau TER was reached, plant lectins or media alone were added in duplicate wells, and the TER was measured at 30 min and 2, 4, 9, and 24 h. The epithelial resistance was expressed as follows :

epithelial resistance = (Ω/cm^2^) - the resistance of transwells without cells.

### HIV cell-free particles transcytosis

the epithelial cell line HEC-1A was grown as a tight polarized monolayer on a permeable support of 0.4-μm-pore-diameter polycarbonate (Transwell), as previously described [10]. The tightness of the monolayer of HEC-1A cells was monitored by measuring resistivity above 200 Ω/cm^2^. HIV-1 (5 ng of p24 antigen) was added together with increasing doses of each plant lectin or mannan (100 μg/ml) on the apical side of the HEC-1A monolayer. Cells were incubated for 1 h at 37°C. HIV-1 transcytosis was assessed by detecting the presence of p24 antigen (HIV-1 core profile ELISA) in the basolateral chamber of the transwell. The inhibition of transcytosis was expressed as the percentage of p24 antigen recovered in the basolateral chamber in the presence of each plant lectin by comparison to that recovered without the plant lectins.

### HIV-1 attachment assay on epithelial cells

HEC-1A cells, seeded at confluency in 48-well plates, were incubated with increasing doses of each lectins or mannan (100 μg/ml) and HIV-1 (5 ng p24 antigen). Each sample was performed in duplicate. After 1 h of incubation, unattached virus was removed (4 washes) and cells were lysed (1% Triton X-100 for 45 min at 37°C). Cell lysates were harvested and centrifuged at 1,800 rpm for 5 min. The amount of HIV-p24 antigen associated to cell lysates was determined using the HIV-1 p24 core profile ELISA.

### HIV-1 attachment on MDDC

to assess the attachment of HIV-1 to MDDC, the cells were washed 2 times after 6 days of differentiation and seeded into 96-well culture plates (1 × 10^5 ^cells/well). HIV-1 (1 ng p24 antigen) and increasing doses of each plant lectin or mannan (100 μg/ml) were added on cells and incubated for 1 h at 37°C in a 5% CO_2 _atmosphere. Each sample was performed in triplicate. After 4 washes to remove the unattached virus, cells were lysed by incubation for 45 min at 37°C with 1% Triton X-100. Cell lysates were harvested and centrifuged at 1,800 rpm for 5 min. The amount of cell-associated HIV-1 was evaluated using p24 antigen capture ELISA.

### MDDC-mediated infection of autologous T cells

to assess the transmission of HIV-1 from MDDC to autologous T-cells, MDDC were incubated into 96-well culture plates (1 × 10^5 ^cells/well) and infected with HIV-1 (0.5 ng p24 antigen) in the presence of increasing concentrations of each plant lectin or mannan (100 μg/ml) for 3 h at 37°C in a 5% CO_2 _atmosphere. Cells were washed four times and autologous stimulated T cells were added onto HIV-exposed MDDC at a DC:T-cell ratio of 1:5. Each sample was performed in triplicate. Culture supernatants were harvested every 3 days and fresh medium was added. Supernatants were inactivated with 1% Triton X-100 and frozen at -20°C. The viral production by T lymphocytes was evaluated at the sixth day of the co-culture by measurement of p24 antigen in supernatants using capture ELISA.

### Confocal microscopy

confluent epithelial cells HEC-1A were trypsinized, washed, and 2 × 10^5 ^cells were adsorbed on a microscopy-adapted slide for 3 days. After 6 days of differentiation MDDC are washed and 2 × 10^5 ^cells were adsorbed on a microscopy-adapted slide. Cells were incubated with or without HHA-TRITC or GNA-TRITC (200 μg/ml; Eylabs) at 4°C for 30 min. Cells were washed with PBS 0.01% azide 0.5% BSA and then fixed with 1% paraformaldehyde. The coverslides were mounted in Mowiol (Sigma-Aldrich). Fluorescence analysis was performed with a Zeiss LSM510 confocal microscope.

### Statistical analysis

unpaired and non-parametric Mann-Whitney U-test was performed for all tests to determine the statistical significance of the data. P < 0.05 was considered the level of statistical significance.

## Results

### HHA and GNA activities in the epithelial cell line HEC-1A

#### (i) Toxicity

**Figure 1:**
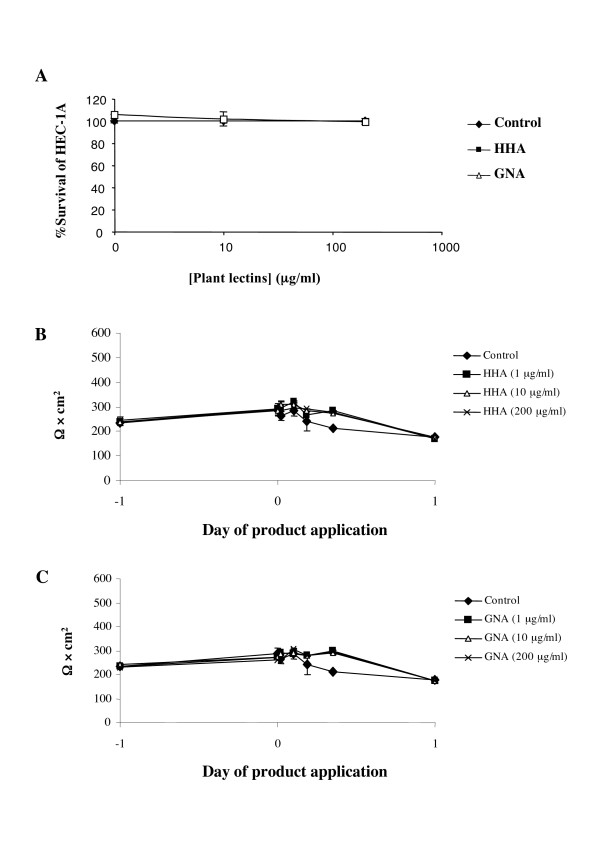
(A). Non-toxic concentration of the study plant lectins in epithelial cell line (HEC-1A) and primary immune cells (MDDC and MDM). HEC-1A and primary immune cells were cultured with concentrations of products for 24 h. After washing, culture viability was determined by using the MTT-cytotoxicity assay. The values given are the percentage of viability. (B) and (C). Effect of HHA and GNA on HEC-1A epithelial monolayer integrity. HEC-1A cells were grown on transwell supports and allowed to form intact monolayer (5 to 7 days). Non-toxic concentrations of each lectins were added to the apical side of the transwell, and epithelial integrity was measured at 30 min and 2, 4, 9, and 24 h by using the Minicell-ERS instrument. The data shown are the averages from duplicate wells ± 1 standard error of the mean after product addition.

Any topical microbicide approved for human use first need to be evaluated for epithelial toxicity, because of the direct contact between the product and the vaginal mucosal surface. Thus, non-toxic concentrations of the plant lectins were determined by culturing the epithelial cell line, HEC-1A, for 24 h in the presence of different lectin concentrations (1 μg/ml to 200 μg/ml) or medium (Fig. 1A). Plant lectins concentrations that gave culture viabilities of more than 80% compared to control cultures were considered to be non-toxic and were further evaluated. Overall, the products exhibited no measurable toxicity towards HEC-1A epithelial cells within a tested concentration range of 1 to 200 μg/ml.

#### (ii) Epithelial monolayer integrity

The ability of mucosal epithelial cells to maintain an intact polarized monolayer in the presence of the plant lectins is a possible predictor of the safety of products on vaginal tissues. Thus, HHA and GNA were added to confluent HEC-1A cell-monolayers, as determined by TER = 200 Ω/cm^2^. The TER was evaluated at 24 h after addition of the plant lectins. HHA up to 200 μg/ml (Fig. 1B) and GNA up to 200 μg/ml (Fig. 1C) did not measurably affect the integrity of the epithelial barrier. Of note, the TER of the control cultures, without the plant lectins, did not vary more than 25% over the 24 h period.

#### (iii) Effect of plant lectins on HIV-1 attachment on HEC-1A

**Figure 2:**
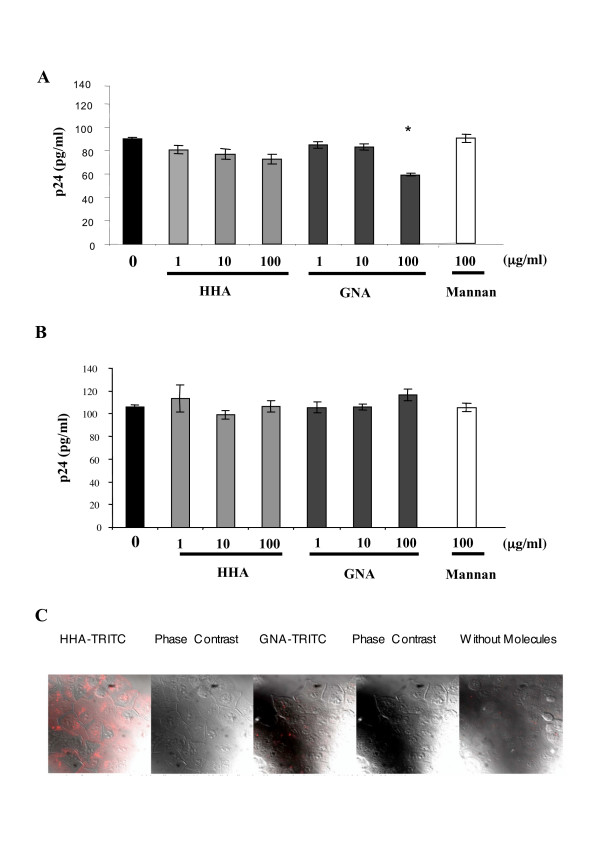
Ability of lectins to inhibit attachment of HIV-1 cell-free particles on epithelial cells. HEC-1A cells were co-incubated with non-toxic concentrations of each lectins or mannan (100 μg/ml) and 5 ng of HIV-1_Ba-L _(A) or HIV-1_NDK _(B) virus were added for 1 h. Incubation with mannan was used as control. The quantity of attached virus was evaluated by measurement of p24 antigen by ELISA. Means of three independent experiments are presented ; error bars represent standard deviations. (C) HEC-1A cells were incubated with or without each labelled-lectin at 4°C. Cells were then analyzed by confocal microscopy. * < 0.05.

Considering that both HHA and GNA were non-toxic in the epithelial cell system, we further investigated the inhibition of HIV adsorption on the apical side of HEC-1A by the compounds. Cells were thus incubated with HIV-1_Ba-L _in the presence or absence of different concentrations of HHA or GNA. As depicted in Figure 2A, HHA faintly decreased the attachment of HIV-1_Ba-L _on epithelial cells (range: 12–17%). Interestingly, GNA afforded a 33% decrease of HIV adsorption only at a concentration of 100 μg/ml. In order to evaluate whether the observed effects of the plant lectins are linked to molecules implicated in HIV attachment towards genital epithelial cells, such as glycosaminoglycans (GAG) heparan sulfates, we assessed the role of plant lectins on the attachment of X4-tropic viruses, known to interact very efficiently with heparan sulfate [11] and data not shown]. Thus, cells were incubated with HIV-1_NDK _(Fig. 2B) in the presence or absence of different concentrations of HHA and GNA. As depicted in Figure 2B, none of the plant lectins, whatever the concentration, inhibited HIV-1_NDK _attachment on HEC-1A cells. These data suggested that HHA and GNA did not interact with HIV recognition sites on GAG. In addition, mannan (100 μg/ml) did not decrease HIV adsorption on HEC-1A cells whatever the viral strain used (Fig. 2A and Fig. 2B). To investigate the efficiency of each lectin to interact with epithelial cell's receptors, labeled HHA and GNA were incubated with epithelial cells. As depicted in Figure 2C, only HHA but not GNA interacted with receptors expressed at the surface of HEC-1. Moreover, the preincubation of GNA with epithelial cells followed with several washes did not inhibit viral attachment whereas the preincubation of GNA with R5 viral particles induced an inhibition of 35 ± 5% of R5 HIV-1 attachment on epithelial cells (data not shown), similarly to the experimentation made without preincubation. Altogether these results suggest that GNA interrupts the virus attachment process by interfering with the virus envelope glycoprotein.

#### (iv) Efficacy of plant lectins on HIV-1 transcytosis through a tight epithelial cells monolayer

**Figure 3:**
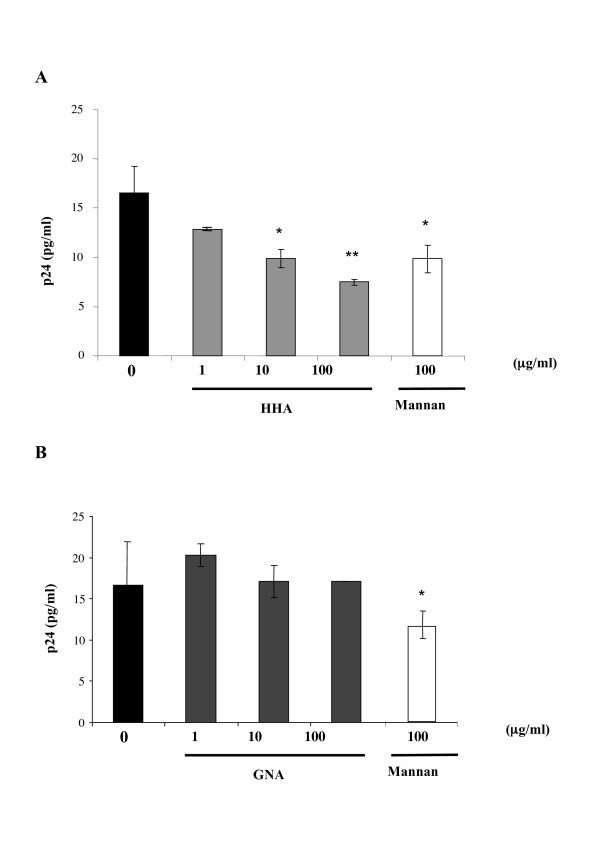
Ability of lectins to inhibit transcytosis of HIV-1_J-RCSF _free particles through a tight monolayer of endometrial epithelial cell (HEC-1A). HIV-1_J-RCSF _(5 ng p24 antigen) was co-incubated with a non-toxic concentrations of each lectins or mannan (100 μg/ml) on the HEC-1A apical pole cultured in dual-chamber by the transwell system for 1 h. Results of an experiment performed in duplicate are expressed as the quantity of virus recovered in the basal chamber in the presence of HHA (A) and GNA (B), or in the absence of lectins. Means of three independent experiments are presented ; error bars represent standard deviations. *< 0.05, **< 0.01.

To evaluate the capacity of each plant lectin to inhibit *in vitro *HIV-1 transcytosis through genital epithelial cells, such as HEC-1A cells, we used a dual-chamber model in which the apical chamber consisted of a confluent monolayer of HEC-1A cells, and the basal chamber contained fresh medium. Cell-free virus (HIV-1_J-RCSF_) was deposited on the apical surface of HEC-1A cells with increasing concentrations of the plant lectins. The HIV-1_J-RCSF _strain was used in this experiment due to the low transcytosis ability of HIV-1_Ba-L _strain [8]. As shown in Figure 3A, HHA inhibited transcytosis of cell-free HIV-1 in a dose-dependent manner. Similarly to mannan (100 μg/ml) that inhibited HIV transcytosis up to 41%, increasing concentrations of HHA (range 1-10-100 μg/ml) afforded a 17%, 42% and 54% decrease of HIV-1_JRCSF _transcytosis, respectively (Fig. 3A). In contrast, GNA did not inhibit the transcytosis of HIV-1 cell-free particles (Fig. 3B). Altogether, these results suggest that HHA inhibited transcytosis by interacting specifically with HEC-1A receptors involved in the HIV transcytosis process [12].

### HHA and GNA activities in monocyte-derived dendritic cells

#### (i) Toxicity

**Figure 4:**
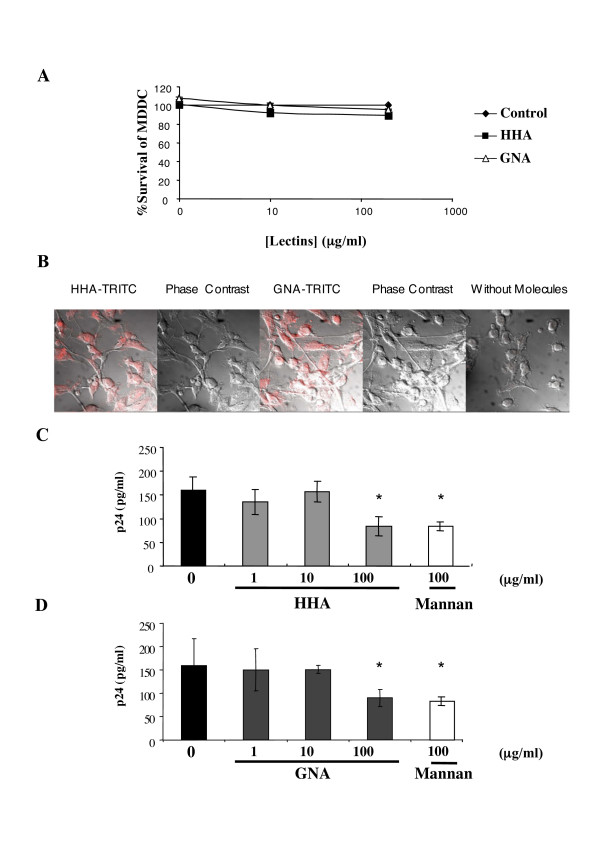
(A). Lack of toxicity of HHA and GNA towards dendritic cells (MDDC). MDDC were cultured with concentrations of products for 24 h. After washing, culture viability was determined by using the MTT cytotoxicity assay. The values given are the percentage of viability. (B). Interactions of plant lectins with surface receptors of dendritic cells. Dendritic cells (MDDC) were incubated with or without each labelled-lectin at 4°C. Cells were then analyzed by confocal microscopy. (C and D). Ability of lectins to inhibit uptake of HIV-1_Ba-L _cell-free particles on dendritic cells. MDDC were co-incubated with non-toxic concentrations of HHA (C), or GNA (D) or mannan (100 μg/ml) and 1 ng p24 antigen of HIV-1_Ba-L _were added for 1 h. The quantity of attached-virus was evaluated by measurement of p24 antigen by ELISA. Means of three independent experiments are presented ; error bars represent standard deviations. *< 0.05

Cellular viability was determined for MDDC by culturing for 24 h in the presence of serial dilutions of the plant lectins. Plant lectin concentrations that gave culture viabilities of more than 80% compared to control cultures were considered to be non-toxic (Fig. 4A).

#### (ii) Efficacy of lectins against attachment of HIV-1 on MDDC

Intraepithelial and submucosal dendritic cells (DCs) and CD4^+ ^T lymphocytes are the predominant cell populations firstly targeted by HIV-1 [13]. Indeed, successful transfer of virus across epithelial barriers would result in HIV-1 capture by DC and subsequent transmission to nearby CD4^+ ^T cells or dissemination to draining lymph nodes. We first investigated whether the plant lectins are able to interact with receptors expressed at the surface of monocyte-derived dendritic cells (MDDC). As depicted in Figure 4B, both HHA and GNA interacted with receptors expressed at the surface of MDDC, suggesting that these plant lectins would interrupt the virus attachment process by interfering both with the virus envelope glycoprotein and with the receptors expressed at the surface of MDDC. Then, we evaluated the effect of plant lectins on HIV-1 uptake by MDDC. Therefore, MDDC were incubated with HIV-1_Ba-L _in the presence of increasing doses of each plant lectin (Fig. 4C and Fig. 4D). Similarly to mannan (100 μg/ml) that induced a 46% decrease of HIV adsorption on MDDC, both HHA and GNA (100 μg/ml) inhibited the virus attachment on MDDC, of 47.2 ± 5.5% and 43.1 ± 5.8%, respectively. No inhibition of HIV adsorption on MDDC was observed for both lectins at lower concentrations (1 and 10 μg/ml).

#### (iii) Efficacy of the plant lectins on HIV-1 transmission to T cell blasts by MDDC

**Figure 5:**
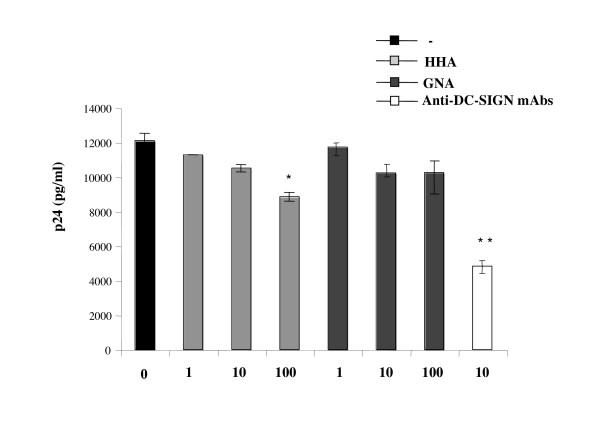
Ability of plant lectins to inhibit transfer of HIV-1_Ba-L _free particles by dendritic cells to autologous T lymphocytes. Dendritic cells (MDDC) were incubated with non-toxic concentrations of each plant lectin and 0.5 ng p24 antigen of virus were added for 3 h. The HIV-production by T cells was evaluated on the 6^th ^day of co-culture by measurement of p24 antigen by ELISA. Means of three independent experiments are presented; error bars represent standard deviations. *< 0.05, **< 0.01.

We finally evaluated whether inhibition of attachment on MDDC resulted in subsequent inhibition of HIV-1 transmission to autologous T lymphocytes. MDDC were incubated with HIV-1_Ba-L _in the presence of increasing doses of each plant lectin. Free viral particles and plant lectins were removed and autologous PBL were added. The pretreatment of MDDC by each plant lectin resulted in a partially reduced infection of autologous PBL by HIV-1 (Fig. 5). We observed 26.9 ± 0.5% of inhibition of HIV transfer at a concentration of 100 μg/ml of HHA and 15.3 ± 1.3% of inhibition at a concentration of 100 μg/ml of GNA. In these same experiments, we used blocking anti-DC-SIGN mAb (clone 507) as positive control of inhibition of transfer [14]. The addition of anti-DC-SIGN mAb inhibited more than 43.8 ± 5.7% both R5- and X4- HIV-1 transfer from MDDC to T cells (Fig. 5).

## Discussion

The plan lectins HHA and GNA constitute two promising microbicide molecules of great interest, capable to efficiently inhibit the infection of T lymphocytes and PBMC by a broad range of HIV strains [4]. This effect is mostly due to direct interactions between the plant lectins and mannose residues at the gp120 of the HIV-1 envelope and thus inhibiting fusion rather adsorption of HIV [9]. However, anti-HIV effects resulting of interactions between plant lectins and cellular HIV receptors were not profoundly investigated. In addition, no direct interaction between the plant lectins and CD4, CCR5, CXCR4 and DC-SIGN was observed [4]. In the present study, we thus aimed at characterizing whether interactions exist between mannose-specific plant lectins and mannosylated HIV-interacting molecules expressed at the cell surface. Indeed, HIV was described to interact with a large number of molecules onto the cell surface during the adsorption phase [2,15-19]. If some of these receptors are mannosylated close to the HIV-binding sites, the plant lectins may recognize such mannosylated residues, and may mask the HIV binding site by steric hindrance, thus limiting HIV attachment onto target cells. Therefore, we particularly focused on the initial phase of virus adsorption.

We evaluated the effect of the plant lectins on HIV adsorption on genital epithelial cells. We found that HHA faintly inhibited attachment of the R5-tropic HIV-1_Ba-L _strain, in a dose-dependent manner, whereas GNA moderately inhibited HIV adsorption in the same context, and only at high drug doses. HSPG are known to be highly expressed on HEC-1 cells [12], and are required for HIV-1 attachment to epithelial cells [20]. HSPG interact with positively charged-V3 loop of X4-tropîc HIV-1 gp120, and weakly with less positively charged R5-tropic strains on epithelial cells [12]. In our hands, GNA did not inhibit the attachment of X4-tropic HIV-1 on epithelial cells, and did not interact with epithelial cell surface. Thus, the preincubation of GNA with epithelial cells did not inhibit viral attachment whereas the preincubation of GNA with R5-tropic HIV-1 particles induced the inhibition of HIV-1 attachment on epithelial cells, suggesting that the antiviral activity of GNA is likely related to its interaction with virus particles. Indeed, altogether our results suggest that HIV-1_Ba-L _interact with (a) membrane molecule(s) expressed on apical side of epithelial cells during the attachment phase which is (or are) (i) not an HIV-interacting GAG, as indicated by our observations in the presence of X4-tropic viruses, (ii) not C-type lectin(s), considering the absence of effect of mannan, (iii) α(1–3) mannosylated close to HIV-binding site, as indicated both by the lack of inhibitory effect on HIV adsorption in the presence of HHA and by the interaction of HHA with epithelial cells receptors. The identification of this (or these) molecule(s) remains to be performed.

We further assessed the role of both plant lectins on HIV transcytosis. Hocini and *al*. have previously demonstrated a partial inhibitory effect of mannan in transcytosis [8], thus involving a C-type lectin, conversely to adsorption phase. Only HHA, but not GNA, inhibited HIV-1_JR-CSF _transcytosis in a dose-dependent manner. Whereas GNA has a specificity for terminal α(1–3)-linked mannose residues, HHA recognizes both terminal and internal α(1–3) and α(1–6)-linked mannose residues [9]. Whether this additional specificity of HHA is the reason for the effect of this plant lectin on transcytosis remains to be demonstrated. Since HHA inhibited specifically transcytosis and faint to inhibit HIV-1 adsorption, we confirm herein that adsorption and transcytosis of HIV are likely two distinct phenomenons involving in the latter one (a) C-type lectin(s) and HHA-interacting mannosylated molecule(s) [12]. Involvement of a single HIV-interacting molecule exhibiting mannosylation close to the C-type lectin region (CLR domain) within the transcytosis phase is also possible. HEC-1 epithelial cells used in our model do not express mannose receptor [19] nor DC-SIGN molecule [15], which could be involved in cell-free HIV-1 particles endocytosis (data not shown).

We evaluated the role of lectins on HIV adsorption on MDDC, representing one of the primary HIV target cells in the course of sexual transmission [13]. Similarly to MDM, HIV is adsorbed on MDDC *via *C-type lectins [15], syndecans [20], and to some extent CD4 [21]. However, the diversity of C-type lectins expressed on MDDC is broader to that expressed on MDM, as illustrated by the restricted expression of DC-SIGN or DEC-205 on MDDC [22,23]. Nevertheless, if some C-type lectins interact with HIV, recent data suggest a specific role for each HIV-interacting C-type lectins. Thus, the mannose receptor seems to be more involved in HIV attachment and internalization whereas DC-SIGN seems to be more involved in the transfer of infectious viruses to susceptible CD4^+ ^T cells [24]. In that context, our results indicated that both plant lectins partially inhibited HIV attachment on MDDC at the highest concentration tested (100 μg/ml), similarly to mannan. Therefore, these results strongly suggest that : (i) both HHA and GNA interact with MDDC surface proteins exhibiting terminal α(1–3)-mannosylation ; (ii) some of these mannosylated proteins interact with HIV in a manner which could be inhibited by plant lectin interaction, and (iii) that some of these mannosylated proteins could be C-type lectins, considering the blocking effect of mannan and the relative role of those lectins, syndecans and CD4 in HIV adsorption on MDDC. Interestingly, a recent report has described a selective tissus-specific mannosylation of the mannose receptor on macrophages which could react in some cases with GNA [25]. Thus, it is also tempting to suggest that the mannose receptor could be mannosylated close to the HIV binding site when expressed on MDDC, conversely to an unmannosylated form of the mannose receptor expressed on MDM, therefore explaining the observed difference between the plant-lectin effects on MDDC and on epithelial cells.

Finally, we assessed the role of HIV-lectins pre-treatment on MDDC to T cells transfer of HIV. Our results showed a modest decrease of HIV transfer between MDDC and PBL. In addition, this down-modulation was inferior to that observed for HIV adsorption on MDDC in the presence of lectins. In the system we used, unbound plant lectins were eliminated by washing treated-MDDC before the addition of T cells, in order to focus exclusively on the effect of adsorbed plant-lectin on MDDC. Our observations suggest that plant lectins act on MDDC more specifically on adsorption than on the transfer phase. This weak efficiency of plant lectins to inhibit HIV-1 transfer was also reported in a recent published study which was performed using cell lines (Raji/DC-SIGN cell system and C8166 T cell line [5]. Recently, it was reported that dendritic cells expressed a large array of receptors implicated on the HIV-1 attachment and/or transfer [2]. In particular, Turville and colleagues reported that dendritic cells do not express DC-SIGN but mannosylated receptors in tissues-where HIV-1 is captured by dendritic cells-, whereas in lymph nodes-where transfer to T cells occurs- dendritic cells express largely DC-SIGN, suggesting that receptors involved in attachment and transfer are different [2]. In the present study, HHA inhibited as efficiently as GNA HIV-1 adsorption on dendritic cells, but only HHA inhibited HIV-1 transfer from MDDC to T cells, suggesting that HIV-1 receptors invloved in HIV attachment and in HIV-1 transfer may be different, and that HHA and GNA may interact with different(s) receptor(s).

Our observations demonstrate furthermore higher inhibitory activities of the lectin plant HHA by comparison to GNA, on HIV adsorption to HEC-1A cell line, HIV transcytosis through HEC-1A cell line monolayer, HIV adsorption to MDDC and HIV transfert from MDDC to T cells, highlighting the potential interest of HHA as effective microbicide against HIV. These findings may have high relevance to be taken into account for the choice and development of microbicide molecule candidates, and likely suggest to retain for further investigations the microbicide molecule candidate the most effective to inhibit HIV in various *in vitro *systems mimicking the attachment of the virus to mucosal target cells and the transport of HIV through the mucosa. Combination products could in principle provide a greater degree of protection than single agents, a broader spectrum of activity against various pathogens and a lower risk of adverse reactions. Since high concentration of HHA are necessary to inhibit the adsorption on the HIV target cells, the combination of HHA derivates with one or two other component(s) would allow a lower dose of this component.

## Competing interests

The author(s) declare that they have no competing interests.

## Authors' contributions

HSand NNcarried out differentiation and infection of dendritic cells, isolation of T cells, HIV-1 transfer assays, cytotoxicity assay, evaluation of the epithelial monolayer integrity and ELISA.

MAJ carried out cytotoxicity assay, transcytosis assays, HIV-1 attachment assays.

ML andCK carried out the confocal microscopy assays and helped draft the manuscript.

JBand DS: provided plant lectins, participated in the design of the study, and helped draft the manuscript.

LB and HS: conceived the study, participated in its design and coordination, and helped draft the manuscript.

All authors read and approved the final manuscript.
